# Medical Opinion and Movement

**Published:** 1909-05-29

**Authors:** 


### Medical Opinion and Movement.

Raymond, in *La Revue d'Obstétrique et de Gynécologie*, advises that in all cases of Acute Puerperal Infection in which there is evidence that the bacterial invasion has spread beyond the uterine walls, the operation of posterior colpotomy should be performed. The technique is simple, the operation itself is slight, and it may be undertaken very early in the disease. Both vaginal hysterectomy and laparotomy are dangerous when the infection is grave, because of the shock which their performance entails. Colpotomy is also useful at the outset in cases of acute pelvic peritonitis, since it is always possible at a later period to resort to more drastic measures. In many cases the operation brings to light peritoneal collections which were unsuspected clinically. Where there is perforation of the puerperal uterus, and more especially when the lesion is on its posterior surface, colpotomy allows of evacuation of the septic fluids and thus prevents general infection. It is often successful in cases in which the infection leads to no peritoneal effusion, and the author considers it absolutely necessary when abscesses are present in Douglas' pouch and the broad ligament. Peri-uterine phlebitis with abscesses should be treated by colpotomy, and it is possible in some cases to remove thrombosed veins by this route. When, however, the collections of pus are large, and there is risk of extension to the general cavity, the operation by itself is not sufficient, but should be combined with drainage through the abdominal wall.

---

One Thousand cases of Serious Head Injury personally observed is a very large number; such a series is analysed by Dr. Charles Phelps in the *Annals of Surgery*. The first point he deals with is the percentage of recoveries, which, though larger than might be expected from some of the statements in text-books, is about what any surgeon of experience would expect to find. Out of 570 cases of fracture of the cranial base there were 259 recoveries, or 45.4 per cent.; out of 213 of fracture of the cranial vault there were 152 recoveries; and out of 217 of independent injury, in which no evidence of fracture could be found (though doubtless many fractures were in fact present), there were 130 recoveries. Another conclusion reached is that hæmorrhage from the ear after head injury, unless it can be proved to come from a lesion of the meatus itself, always means fracture of the petrous bone, whether the tympanum has or has not been damaged: it is impossible for concussion or other violence to rupture the tympanum without also fracturing the temporal bone. In 90 cases the only exception to this was in the case of a gunshot wound at close range. Linear fracture practically always unites by callus: displacement is extremely rare, but does occur. A definite loss of osseous substance, as by the complete removal of a detached fragment, is replaced by a dense fibrous structure composed of the thickened and consolidated dura mater and periosteum. The prognosis of a depressed fragment depends on treatment: with early elevation it is very good. The only danger due to fracture itself, as distinguished from concomitant brain injury, is that of laceration of vessels or of cerebral tissue.

---

As regards treatment, Dr. Phelps formulates a procedure which has, he believes, been justified in his practice, as well as by theoretical considerations. During the continuance of shock no local interference is permitted except for hæmorrhage or for the aseptic protection of a compound fracture. As soon as practicable after reaction begins, examination, and if necessary thorough exploration for fracture, are made. If any suspicion of fracture exists, exploratory incision is performed even in the absence of symptoms of cerebral lesion. A fine fissure is neglected, but an open one entails further exploration for comminution or depressed inner table. Punctured fractures are explored when attended by symptoms. All depressed or comminuted fragments are carefully elevated, but nothing is removed unless it has no dural or periosteal attachment worth considering. Trephines are avoided whenever it is feasible to manage with chisel, drill, or rongeur alone. Direct fractures of the base, and the basal continuations of fractures of the vault rarely admit interference; exceptions admitted are in orbital and ethmoid fractures, due to direct violence. The antiseptic treatment of a ruptured tympanum and petrous portion is regarded as excellent theoretically, but seemingly not as of any great practical moment. When Dr. Phelps comes to the consideration of the after results in those 541 patients who recovered, it may be anticipated that he will have some even more interesting conclusions to publish.

---

Two very vexed questions in the Early Treatment of Appendicitis are discussed by Mr. A. E. Maylard in the *Practitioner*. These two conundrums have divided the profession ever since the recognition of appendicitis became general near the end of the last century, and its operative treatment was admitted and discussed. Mr. Maylard puts them thus: Should aperients or sedatives be administered in the early stages of the attack? and Should heat or cold be applied locally? These two queries he discusses (apart altogether from questions of operation) in their theoretical and practical aspects. To the first he answers that he would allow $\frac{1}{6}$ grain of morphia for pain solely, and would commence at once the administration of drachm doses of sulphate of magnesia every hour until the bowels move. As to the second he prefers hot fomentations to ice-bags, applied to the iliac region. The author recognises that theory takes a more prominent place than facts in the details of his discussion of the problem, a position to a large degree unavoidable in our ignorance of the exact processes at work in any particular case, and of the influences exercised by any particular agent. Probably very many surgeons would disagree with his opinions, especially the first one; but these divergences of teaching serve to emphasise the protean character of the disease and the fact that finality is by no means yet reached about its correct treatment.

THE claims of treatment, if this term is taken to include general management and control, as well as more active obstetrical measures, are usually so insistent in midwifery work, that the hard-pressed general practitioner is often tempted to scamp his routine determination of the exact fœtal presentation, etc. Indeed, there are country practitioners of considerable skill and great experience and success in practical obstetrics who habitually neglect the precise academical diagnosis so much beloved of examiners and teachers, and who yet seem to get along very well without it. Nevertheless they are bad examples to the young practitioner, for their violation of one of the canons of the art is often more apparent than real, diagnosis with them being rapid and intuitive; in any case their success is obtained at a cost, and would be greater and of a higher type if their knowledge of the conditions under their eyes were more precise and self-conscious. Therefore it is not time wasted upon mere academic refinements for the practitioner to make a note of the simple and practical method described by Kocks for the diagnosis of the fœtal lie in Shoulder Presentations. One arm is drawn down and the hand pulled out of the vagina. The limb is then placed in a position of extreme supination—*i.e.* with the thumb as near as possible to the back of the hand. The thumb then points to the side of the mother on which the child's head lies, the back of the hand corresponding to the back of the fœtus. If the back of the hand is in front the back of the fœtus is anterior, and the presentation is occipito-anterior; if the back of the hand is behind the back of the fœtus is posterior, and the presentation is occipito-posterior. If the thumb points to the mother's right, the head lies to the right; if to the mother's left, the head lies to the left. When the diagnosis has been made, the limb is returned to the uterus and version performed.

---

IN such affections as Chlorosis, Early Tuberculosis, etc., the patient's anæmia and her diminished resistance to the disease are often increased by over-frequent menstruation. With the idea of checking this most debilitating symptom Wilczinski, of Zakopane, has tried the effect of the administration of lecithin in doses of 0.1 to 0.2 gram three times a day, commencing immediately on the cessation of a period. The author has been able by its use to delay the appearance of the flow for a week on two consecutive occasions in the same patient, who, at the outset of this treatment, fifteen months ago, menstruated twice a month. On the third occasion an interval of four weeks occurred, and since then the patient has continued to menstruate with intervals of four weeks. Equal success attended the case of a woman who, though regular, suffered from excessive hæmorrhage at the periods. Here, under lecithin, the period was twice delayed for two weeks. Out of 21 patients so treated eight were much improved, one failed to react to treatment, and the remaining 12, though improved, have not been under treatment sufficiently long to judge of its results. The author points out the necessity of persevering with the drug for a long time during inter-menstrual periods. A prolonged course of lecithin presents no dangers to the patient.

DR. C. F. HEERFORDT, of Copenhagen, gives an account of three cases he has observed within the past few months of a condition which appeared in many respects to suggest an atypical form of Mumps. But, on the other hand, these cases took such an unusual clinical course that he is induced to regard the condition as a separate, unknown morbid entity. The patients were 11, 14, and 27 years of age respectively. The malady commenced with prodromata—anorexia, epistaxis, and malaise—which lasted from 14 days in one case to three months in another. Then followed the development of the characteristic symptoms—namely, fever, irido-cyclitis, enlargement of the parotid glands, and paresis of the cerebro-spinal nerves. In one case the symptoms developed almost simultaneously, in the second case the disease commenced with a bilateral irido-cyclitis and optic neuritis, and the parotitis developed three weeks later. The third case started with the swelling of the parotid gland, ocular troubles appearing about a month later, to be followed three months later by troubles in deglutition and speech, and paræsthesia of the left side. The peculiar characteristic of this condition, as distinguished from mumps, is the slow subchronic development of the disease, extending in one case over several months. Dr. Heerfordt would distinguish it by the name of sub-chronic uveo-parotidian fever. He finds in medical literature two cases recorded of an unusual form of mumps which appear closely similar to these cases observed by himself. One was reported by Dr. P. Daireaux, of Paris, in 1899, and the other by Dr. A. Collomb, of Geneva, in 1903. In these cases the parotitis was associated with irido-cyclitis and facial paralysis, and the course of the disease appears to have been prolonged over several weeks.

---

FLUORESCENT substances (such as fluorescin, eosin, acridin, and scarlet red) have been known to exercise under the influence of sunlight a bactericidal effect. Von Toppeiner has reduced the power of diphtheria toxin by exposure to eosin and sunlight, and he has obtained good results by applying it to parasitic affections of the skin and afterwards exposing the parts to sunlight. Noguchi has obtained similar experimental results with animals. More recently Drs. V. and W. Pleth, of Guadalajara, Mexico, have obtained good effects by treating artificially-produced ulcerations in animals with a 5 to 10 per cent. aqueous solution of eosin and with scarlet red suspended in olive oil or vaseline, and exposing to sunlight. The animals treated with eosin recovered rapidly, while some of the control animals even died from the infected wounds. These favourable results led to the adoption of the treatment in all manners of lesions in the human subject, and a report of their work appears in the *American Journal of Surgery*. The authors have used the treatment in cases of suppurating wounds, infected joints, eczema, actinomycosis, endometritis, and other conditions. They have frequently used the two substances alternately, painting the part one day with eosin and the next applying the scarlet red suspended in oil or vaseline, and they are much impressed with the rapidity of healing under this method of treatment. The chief drawback is the staining of the clothes.

Dr. G. Pochou makes some interesting observations in the *Journal de Médicine* on Mammary and Ovarian Opotherapy and the antagonism between the secretions of the mammary and ovarian glands. The author cites several cases of severe hæmorrhage at the menstrual periods, which he has been able to control successfully by the administration of mammary gland in cachets. Even in cases in which the presence of a small fibroid is a causal factor, chiefly, he thinks, by the general uterine congestion which it produces, considerable relief is obtained by the administration of mammary gland. He finds, moreover, that the administration of the gland often causes a swelling and activity of the breasts, and may cause a return of proper lactation in cases in which a diminution of milk secretion is attributable to a return of the menstrual periods. On the other hand, the author reports several cases in which suppression of the menses has been accompanied by swelling and tenderness of the breasts, and in such cases the administration of ovarian gland has effected a return of the menstrual periods, together with a reduction of the breasts to normal conditions. These cases afford not only a therapeutic interest, but also suggest a physiological antagonism between the mammary and ovarian glands. The author points out that this antagonism may assert itself especially at the menopause, when a deficient ovarian activity may result in a congestion of the breasts, and he suggests that this congestion may favour the development of neoplasms, which make their appearance especially at this age. Velpeau has drawn attention to the fact that adenoid tumours of the breast are frequently associated with irregularity of the menstrual functions.

---

During the past year considerable attention was devoted to the danger of Typhoid Bacilli Carriers, and on several occasions, especially in asylums and like institutions, continued outbreaks of the disease have been traced to this source. They are discovered by a careful bacteriological examination of the fæces, and it has been almost conclusively shown that the gall-bladder forms the habitat of the bacilli, from which they are extruded into the intestinal tract, and so arrive in the fæces. Even careful examination of the fæces, however, unless constantly repeated, may fail to discover the presence of the bacilli, as they may only be eliminated at intervals. According to Jurgens and Forster, they often exist in large quantities in the upper portion of the intestinal tract, but steadily diminish in their course downwards. Weber, therefore, has conceived the idea of causing a reflex of bile into the stomach in suspected cases, according to the method of Volhard, and examining the bilious gastric content for the presence of the bacillus of Eberth. The patient is given 200 c.c. of olive oil, and half an hour afterwards the gastric content is drawn off. This consists of a liquid which separates into two layers—an aqueous below, which is coloured with bile, and an oily layer which floats on the top. It is in the oily layer that the bacilli are discovered. In this way the typhoid bacilli have been detected in the gastric content in enormous quantities in cases in which the fæces only showed them in sparse colonies. Moreover, these micro-organisms may be identified in the gastric content after an interval of two days, so that the fluid can be sent to a laboratory at a distance for examination. It is not always easy to obtain a reflex of bile into the stomach, but even when the gastric content is only slightly coloured with bile a positive result is said to be obtained in cases of "typhoid carriers."

---

Quintard, in a report on two cases of Tubercular Stenosis of the Ileum, lays stress on the difficulty in correctly diagnosing this condition. "The majority of cases," he remarks, "where one really does not know anything definite about the diagnosis, and where carcinoma and bands can be excluded, prove to be of tubercular origin." In the two cases reported there was a definite history of recurrent abdominal pain, which in one was accompanied with marked visible peristalsis. The pain and discomfort follow the intake of solid food, and very little aid is obtainable from an inspection of the fæces. The condition appears to be commoner in young adult patients than in any other class. A most important detail, from the practitioner's point of view is, of course, the question of treatment. In the discussion which followed the reading of Dr. Quintard's paper at the New York Post-graduate Clinical Society, Erdmann advocated surgical interference in all suitable cases, and thought a lateral anastomosis with resection of the piece of intestine implicated was the most satisfactory method of dealing with the obstruction. The cases he had operated upon have not shown him that there is any validity in the objection that the end pockets, after resection, may become filled up and give rise to secondary trouble after a lateral anastomosis has been performed. Lloyd, again, advised an end to end anastomosis into the ascending colon. In all cases where external signs are wanting the diagnosis presents great difficulties, and where extensive tracts of bowel and mesentery are affected, surgical treatment does not often effect a cure.

---

At the last meeting of the Société de Médecine D'Alger, Vincent showed an Enormous Spleen, which had been removed by resection from a patient who for eighteen months had suffered from what were looked upon as severe abdominal crises. Four years before the operation the patient began to feel vague aches in the left hypochondriac region. These "douleurs vagues" Vincent considered as the first phase in a case of extreme mobility leading on to final torsion. Later on these vague pains were followed by definite signs of displacement of the organ, which could now be felt lying in the right iliac fossa. Finally an acute crisis supervened, accompanied with much vomiting, slight fever, and signs of strangulation. The operation showed that the pedicle was enormously swollen, the veins thrombosed, and that there were areas of thrombosis and incipient gangrene in the gastro-splenic omentum. Notwithstanding this grave complication the patient made a good recovery.

